# A systematic literature review of the assessment of treatment burden experienced by patients and their caregivers

**DOI:** 10.1186/s12877-019-1222-z

**Published:** 2019-10-11

**Authors:** Orla C. Sheehan, Bruce Leff, Christine S. Ritchie, Sarah K. Garrigues, Lingsheng Li, Debra Saliba, Roya Fathi, Cynthia M. Boyd

**Affiliations:** 1 Center on Aging and Health, Johns Hopkins University School of Medicine, Suite 2-700, 2024 E. Monument Street, Baltimore, MD 21205-2223 USA; 2 Division of Geriatric Medicine and Gerontology, Center for Transformative Geriatric Research, Johns Hopkins University School of Medicine, Mason F. Lord Building, Center Tower, 5200 Eastern Avenue, 7th Floor, Baltimore, MD 21224 USA; 3 Department of Health Policy and Management, Johns Hopkins Bloomberg School of Public Health, Baltimore, MD USA; 4 Department of Community and Public Health, Johns Hopkins School of Nursing, Baltimore, USA; 5 Division of Geriatrics, University of California, San Francisco, 3333 California St, Suite 380, San Francisco, CA 94143-1265 USA; 6 VA Quality Scholars Fellowship Program, San Francisco VA Medical Center, 4150 Clement Street, VA181G, San Francisco, CA 94121 USA; 7 UCLA/ JH Borun Center and Los Angeles VA GRECC, 10945 Le Conte Avenue, Suite 2339, Los Angeles, CA 90095 USA; 8 Department of Adult and Family Medicine, Primary Care at Home, Kaiser Permanente San Rafael, 99 Montecillo Road, San Rafael, CA 94903 USA

**Keywords:** Treatment burden, Multiple chronic conditions, Systematic review, Patient, Caregiver

## Abstract

**Background:**

Many older adults with multiple chronic conditions, particularly those who are functionally impaired, spend considerable time juggling the competing demands of managing their conditions often assisted by caregivers. We examined methods of assessing the treatment burden experienced by this population as a first step to identifying strategies to reduce it.

**Methods:**

Systematic searches were performed of the peer-reviewed and grey-literature (PubMed, Cochrane library, CINAHL, EMBASE, Web of Science, SCOPUS, New York Academy of Medicine Grey Literature Review, NLM catalog and ProQuest Digital Theses and Dissertations). After title and abstract screening, both qualitative and quantitative articles describing approaches to assessment of treatment burden were included.

**Results:**

Forty-five articles from the peer reviewed and three items from the grey literature were identified. Most articles (34/48) discussed treatment burden associated with a specific condition. All but one examined the treatment burden experienced by patients and six addressed the treatment burden experienced by caregivers. Qualitative studies revealed many aspects of treatment burden including the burdens of understanding the condition, juggling, monitoring and adjusting treatments, efforts to engage with others for support as well as financial and time burdens. Many tools to assess treatment burden in different populations were identified through the qualitative data. The most commonly used instrument was the Treatment Burden Questionnaire.

**Conclusions:**

Many instruments are available to assess treatment burden, but no one standardized assessment method was identified. Few articles examined approaches to measuring the treatment burden experienced by caregivers. As people live longer with more chronic conditions healthcare providers need to identify patients and caregivers burdened by treatment and engage in approaches to ameliorate treatment burden. A standard and validated assessment method to measure treatment burden in the clinical setting would help to enhance the care of people with multiple chronic conditions, allow comparison of different approaches to reducing treatment burden, and foster ongoing evaluation and monitoring of burden across conditions, patient populations, and time.

**Electronic supplementary material:**

The online version of this article (10.1186/s12877-019-1222-z) contains supplementary material, which is available to authorized users.

## Background

Multimorbidity now affects 65% of older adults in the United States [1]. Many people live a significant portion of their lives with long-term medical conditions that require constant input both from the health care system and from the patient themselves [2]. As the complexity and choice of treatments have grown, many patients and their families struggle to manage the responsibilities and burdens that come with managing multiple chronic conditions. Treatment burden refers to the impact on patient functioning and well-being imposed by the demands on a patient and their caregiver’s time and energy by both treatments and aspects of self-care such as health monitoring, diet and exercise [3].

Treatment burden usually adds to the symptoms and physical and psychological difficulties imposed by the condition itself [4]. Treatment burden is patient specific. For some individuals, burden may be transient in the context of an acute illness and gladly tolerated on a temporary basis in the service of achieving a health care-related goal. For others, the burden of taking multiple oral medications may be accepted, but the challenge of self-administering injections may be too great to overcome [5]. Many tasks on their own appear simple –such as taking new medications, organizing and undergoing tests, and making lifestyle changes. When the number of tasks continues to rise, however, and begins to interfere with work, family and other commitments, the burden of treatment on patients and their families can be very high [6, 7]. Family members often find themselves in the untrained role of informal caregiver learning to give injections, manage polypharmacy or navigate the healthcare system [8]. Too often providers do not recognize the burdens experienced by patients and caregivers and this lack of recognition can contribute to the difficulties of patients to adhere to the provider recommended management of their condition [9].

Person-centered care seeks to minimize treatment burden by tailoring treatment regimens to the realities of the daily lives of individual patients and their particular goals by engaging in Minimally Disruptive Medicine [10]. Reliably and efficiently assessing treatment burden in clinical practice would alert healthcare professionals to patient or caregiver distress, allow healthcare professionals to develop a partnership with the patient, and encourage them to work together to agree upon treatment strategies that are both effective and acceptable for the patient and caregiver. Although multiple studies have documented treatment burden [11] and developed tools to assess the burden, no common approach or assessment instruments have been employed in clinical practice. Importantly, we do not know the extent to which these approaches accommodate measurement of treatment burden in the context of multimorbidity.

We performed a systematic literature review to identify articles or sources assessing the treatment burden experienced by adult patients and caregivers. Our ultimate aim is to develop a treatment-burden-related quality indicator that could work across a range of patients with multiple chronic conditions receiving home-based medical care.

## Methods

### Definition of treatment burden

Building upon previous definitions [3] we defined treatment burden as the effort required by the patient or caregiver to manage the medical conditions of the patient and the impact that this has on their lives. Management includes treatments and self-care or caregiver tasks required to address, treat or monitor specific conditions. Perceived burden varies depending on factors such as available time, additional medical conditions, other responsibilities and treatment related factors such as cost, intensity, difficulty and complexity [12, 13].

### Search strategy

**Fig. 1:**
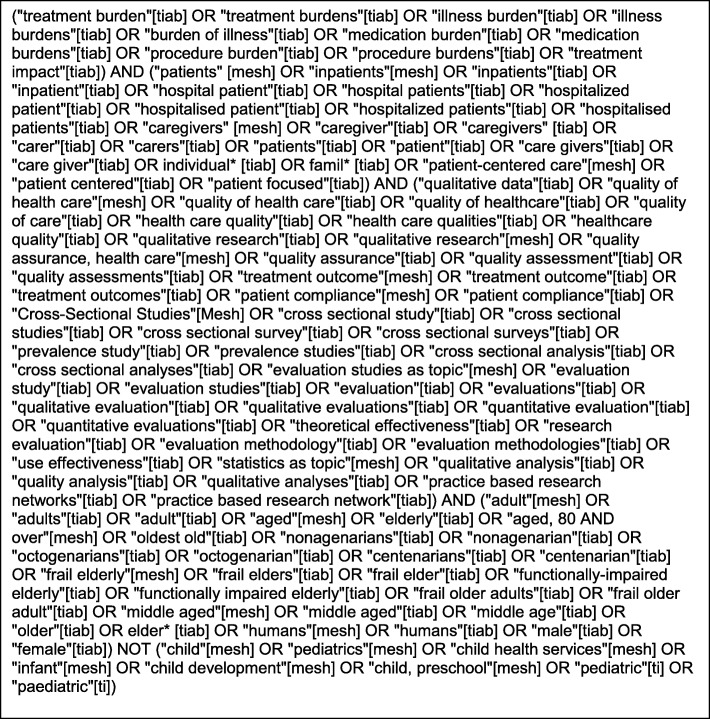
Final pub-med search

The search was conducted in accordance with the PRISMA (Preferred Reporting Items for Systematic Reviews and Meta-Analyses) guidelines [14] and using similar methodology to other systematic reviews performed by our group [15, 16]*.* PRISMA provides an evidence-based minimum set of items for reporting to allow a transparent and complete reporting of systematic reviews. Working with a clinical informationist, an expert in the area of treatment burden (CB) and our research team we identified and refined MESH (Medical Subject Headlines used for indexing articles in PubMed) and keyword search terms related to treatment burden. Sample searches were performed to identify articles deemed highly relevant. After finalizing the search terms, systematic searches were performed of both the peer-reviewed and grey literature. Following the initial PubMed search, search terms were further refined to facilitate searches of the Cochrane library, The Cumulative Index to Nursing & Allied Health (CINAHL), EMBASE, Web of Science, SCOPUS, the New York Academy of Medicine Grey Literature Review, the NLM catalog and the database of ProQuest Digital Theses and Dissertations. The search terms used in the final PubMed search are shown in Fig. 1. Searches were performed for articles relating to humans from 1997 onwards and those in the English language and were performed in March 2019. Similar searches were performed of relevant journals not indexed in Medline. To complete the grey literature review, the first 300 hits from a google search were reviewed for relevance. A search was also performed of relevant guidelines as well as the websites of advocacy groups. Hand searches were conducted of the reference lists of relevant retrieved articles.

### Inclusion, exclusion, and assessment of studies

All articles were entered into reference manager, Endnote v7. Duplicates were removed and the search results were reviewed in a step wise process. First, we screened the titles and abstracts of retrieved articles for relevance to the study aim. Next, full length manuscripts or publications were obtained for any studies that discussed or provided specific recommendations for assessing treatment burden. Articles which referred to any method (qualitative or quantitative) of assessing the treatment burden experienced by patients, caregivers or both for either research or clinical care were included. Articles discussing treatment burden both in general or in relation to one or more specific medical conditions were included. Publications not addressing treatment burden experienced by patients (e.g. only focusing on the overall disease burden, not treatment specific, or the burden of cost on the healthcare system) were excluded as were articles describing treatment burden in pediatric populations. Information on study design, population studied, chronic disease(s), aims, findings, outcomes and measures of treatment burden were extracted from selected articles, publications and guidelines and entered into an excel spreadsheet.

## Results

### Search results

**Fig. 2:**
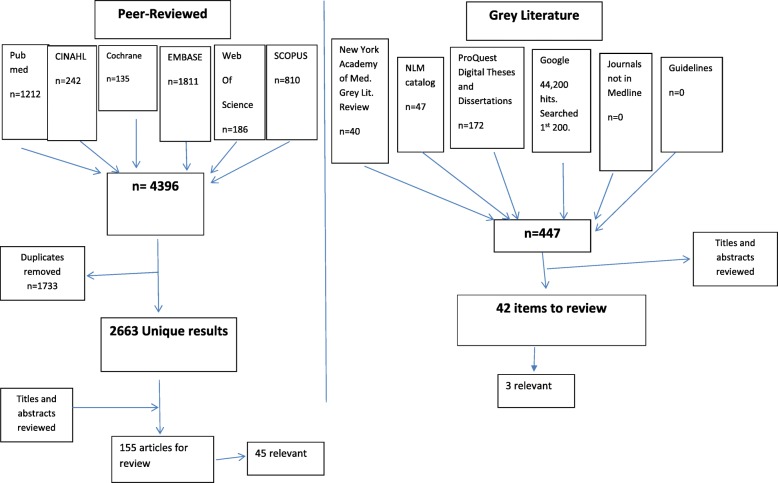
Flow diagram of data sources, search results and process for identification of relevant articles

Following a review of the title and abstracts of 1987 publications, our systematic review of the peer-reviewed literature identified forty-five relevant articles. A further three items (1 book and 2 PhD theses) were identified from a search of 447 titles and abstracts from the grey literature. Forty-eight articles are included in the final sample. The search strategy and results are outlined in Fig. 2, and relevant articles are summarized in Tables 1 and 2 and Additional file 1.

**Table 1:** Qualitative papers exploring the treatment burden experienced by patients and caregivers

Study	Disease	Purpose of study	Specific question / topics asked
[30]	Asthma	Compare the burden of disease and treatment in patients with asthma.	What is your experience of having asthma? How does it affect your life? What is your experience of asthma medicines? How effective are they and do you experience side effects?
[4]	Chronic conditions	Types and consequences of treatment burden.	What are the effects of excessive treatment burden in patients with multimorbidity? How might treatment burden be decreased in patients with multimorbidity?
[22]	Chronic Conditions	Burden of treatment regimens on consumers.	Questions included perspectives on the burden of chronic illness, the burden of treatment and alleviating treatment burden.
[25]	Chronic conditions	Explored treatment burden among people with chronic conditions.	Questions explored the extent and duration of illness, difficulties with medications, finances, relationship with healthcare professionals and daily practical challenges.
[24]	Chronic Conditions	Factors that patients draw on to lessen burden.	How patients cared for their conditions and the impact that care had on them, including their personal life, social situation, and work. Factors that made their care easier or more difficult?
[26]	Chronic conditions	ICAN Discussion Aid	Completed by patients and used during the encounter between patients and health professionals to understand patient capacity, workload, and treatment burden.
[20]	Chronic conditions	Review qualitative literature on burden of treatment	9 studies included. Which components form the burden of treatment in the view of patients with multimorbidity? How is the patient-experienced burden of treatment in patients with multimorbidity conceptualized in the included studies?
[19]	COPD, lung cancer	Review features of treatment burden in these conditions	127 articles included.
[27]	COPD	Explore understanding and experiences of treatment burden	Explored burden from prescribed drug treatment, required health-behavior changes, and interactions with health professionals or health services. Treatment burden was then graded.
[18]	Heart Failure	Review qualitative literature of end-stage heart failure.	16 different articles included
[21]	Heart Failure	Is Normalization Process Theory a useful framework to treatment burden in heart failure?	Questions included how the condition affected the patient’s life at home, ability to perform daily chores, routine, social life/leisure activities as well as if the condition prevented them from doing anything they wish to do?
[28]	Heart Failure	WALT instrument used to assess burden of therapy	Assessed willingness to undergo therapy given the burden imposed by the therapy, the health state, likelihood of the health state and expected life extension, resulting from the therapy.
[29]	CHF, COPD or cancer	Effect of treatment burden on treatment preferences at the end of life.	Questions on a participant’s desire for treatment: the treatment itself, quality-of-life considerations, and the issue of the uncertainty of the outcome.
[38]	Cystic fibrosis	Explore the perceived treatment burden of patients and its correlation with other factors.	To what extent do your treatments make your daily life more difficult? How much time do you currently spend each day on your treatments? How difficult is it for you to do your treatments (including medications) each day?
[23]	Macular Degeneration	Explore the psychosocial impact of repeated intravitreal injections	Semi-structured, one-on-one interviews on treatment burden and satisfaction, tolerability, barriers to adherence, treatment motivation, and patient education.
[31]	PEG	Report the burden of treatment from the patient perspective.	Do you think that having a PEG has changed your life in any way? Is there anything which has been especially difficult, in living with a PEG?
[17]	Stroke	Examine the qualitative literature on stroke and treatment burden.	69 different studies included
[32]	Stroke	Create a conceptual model of treatment burden and patient capacity	Coding framework informed by Normalisation Process Theory (NPT) to organize the patient workload of chronic disease management into the following broad categories: sense-making; interacting with others; enacting management strategies; and appraisal work.

*PEG* Percutaneous endoscopic gastrostomies, *WALT* Willingness to Access Life-Sustaining Treatment, *CHF* Congestive Heart Failure, *COPD* Chronic obstructive pulmonary disease, *ICAN* Instrument for Patient Capacity Assessment

**Table 2:** Quantitative studies assessing treatment burden and the specific instruments or question used

Reference	Disease	Instrument or question used to assess treatment burden
[65]	Asthma	Satisfaction with Inhaled Asthma Treatment Questionnaire
[35]	Bronchiectasis	Quality Of Life -B 3.0 questionnaire
[45]	Cancer (breast)	Quality of life questionnaire
[41]	Cancer (Lung)	Number of encounter days (inpatient or outpatient)
[40]	Cancer (seminoma)	Number of treatment episodes.
[34]	Cancer, congestive heart failure or chronic obstructive pulmonary disease	Willingness to Access life-Sustaining Treatment (WALT) instrument
[44]	Celiac disease	Participants asked to rate 4 domains on a scale of 0–100: difficulty in following treatment, perceived importance of following treatment, disease-specific health and overall health.
[52]	Chronic conditions	Living with Medicines Questionnaire V3 (LMQ-3)
[51]	Chronic conditions	Patient Experience with Treatment and Self-management (PETS)
[57]	Diabetes	
[7, 54]	Chronic conditions	Health Care Task Difficulty.
[47]	Chronic conditions	Treatment Burden Questionnaire (13-item)
[48]	HIV	
[11]	Chronic conditions	Treatment Burden Questionnaire (7-item)
[46, 49, 59]	Chronic conditions	Adapted Treatment Burden Questionnaire (15-item) to include financial burden question and side effects of medication question
[53]	Chronic conditions	Exercise Therapy Burden Questionnaire (ETBQ)
[50]	Multimorbidity	Multimorbidity Treatment Burden Questionnaire (MTBQ)
[38]	Cystic Fibrosis	As part of the CFQ-R, treatment burden was assessed with 3 questions.
[37]	Diabetes	Diabetes Treatment Burden Questionnaire (DTBQ)
[43]	Lupus nephritis	Likert scale rating of treatment burden.
[56]	Medicare beneficiaries	4 questions about things patients are asked to do to stay healthy or treat health problems.
[36]	Psoriasis	Dermatology Life Quality Index
[42]	Stroke	Polypharmacy used as a measure of treatment burden
[39]	Urinary Incontinence	Patient Satisfaction Questionnaire

CHQ: Child health questionnaire; CFQ-R: Cystic Fibrosis Questionnaire – Revised; TBQ: Treatment Burden Questionnaire; Diabetes Treatment Burden Questionnaire: DTBQ; Patient Experience with Treatment and Self-Management: PETS; Living with Medicines Questionnaire V3: LMQ-3; Medicine Regimen Complexity Index: MRCI; Exercise Therapy Burden Questionnaire: ETBQ; Multimorbidity Treatment Burden Questionnaire (MTBQ), Willingness to Access Life-Sustaining Treatment: WALT

#### Population and study setting

Thirty-four of the 48 articles (70.8%) discussed treatment burden associated with specific conditions e.g. heart failure, cancer, cystic fibrosis with the remaining fourteen articles focusing on patients with multiple chronic conditions. Forty-six articles examined treatment burden experienced by patients and six discussed that experienced by caregivers. Three articles [17–19] reviewed the qualitative literature on treatment burden and a chronic condition (stroke, heart failure, respiratory disease) and one article [20] reviewed it in persons with multiple chronic conditions. Quantitative assessment of treatment burden was reported in 30 studies and 18 articles described qualitative approaches.

#### Qualitative studies of assessment of treatment burden

Articles using qualitative analyses of focus group or semi-structured interview data identified the main dimensions of treatment burden experienced by patients [4, 17–22], factors which increase burden [19, 20] and methods by which patients decrease burden [20, 23]. Treatment burden was present both in populations with one specific condition and in those experiencing multiple chronic conditions. In the stroke and heart failure populations the four main dimensions of treatment burden identified through the qualitative literature were coherence (understanding the condition and its implications), treatment and management, appraisal (juggling, monitoring and adjusting treatments) and relationship work (efforts to engage with health professionals, family and patients for help and support) [17, 21]. Table 1.

Specific conditions generated specific burdens, for example, patients with percutaneous endoscopic gastrostomies were most burdened by lifestyle restrictions and practical limitations [31]. The anxiety of receiving intravitreal injections was a source of treatment related burden in macular degeneration [23]. Patients with lung cancer and COPD reported reduced capacity to manage workload due to illness/smoking-related stigma [19]. Many people described the burden of educating themselves about their disease and learning how to self-manage their condition [19, 27, 30, 32]. Patients with chronic conditions and their unpaid caregivers felt that financial burden was the most problematic for them but also felt burdened by time and travel issues, medications and healthcare access [20, 22, 23, 25]. Many treatment related burdens described in relation to a specific condition could also apply to other conditions, for example, multiple myeloma treatment which led to a substantial psychological and physical burden on patients disrupting social activities, decreasing independence and impacting relationships [33].

One group used the Instrument for Patient Capacity Assessment (ICAN) discussion aid during the physician encounter to promote discussion in order to understand patient capacity, workload, and treatment burden [26]. One of the questions in the aid asks “What are the things that your doctors or clinic have asked you to do to care for your health?” Examples are given and patients are asked *“*Do you feel that they are a help, a burden, or both?”

Treatment burden was increased by polypharmacy, multimorbidity, reduced physical, financial and cognitive capacity, barriers to accessing services, fragmented and poorly organized care, lack of continuity of care and inadequate communication between healthcare professionals [19–21]. The same burden was decreased in people with multiple chronic conditions by using problem or emotion focused strategies (e.g. technology, routinizing self-care, enlisting support, maintaining a positive attitude, spirituality), adaptation, prioritization, receiving social support and identifying the positive aspects of health care [20, 24]. Capacity to manage treatment burden was influenced by personal attributes and skills, physical and cognitive abilities, support network, financial status, life workload and environment [32]. Patient preferences were variable based on the substantial differences within the population studied regarding willingness to undergo more or less burdensome therapies [28]. When preferences were tracked over time participants became less willing to endure a high burden of therapy to avoid death [29]. Concern about cognitive disability also rose with time, with participants becoming less willing to accept even low risks of cognitive disability when receiving treatments aimed to prolong life [34].

#### Quantitative studies of assessment of treatment burden

Many instruments and measures used to assess treatment burden were disease-specific (e.g. the Quality of Life-Bronchiectasis (QOL-B) questionnaire [35], the Dermatology Life Quality Index [36] or the Diabetic Treatment Burden Questionnaire [37]). In general, these instruments assessed treatment burden as part of longer quality of life questionnaires [33, 36–39]. Studies describing disease-specific assessments of treatment burden tended to be in younger populations and the conditions studied required specific and time-consuming interventions on the part of the patient e.g. applying creams in psoriasis, respiratory treatments in cystic fibrosis or dietary restrictions in celiac disease. Three studies in cancer patients receiving short term intensive chemotherapy and radiotherapy sessions used number of treatment episodes [40], number of encounter days and number of physicians involved in care [41] as surrogate markers of treatment burden. Polypharmacy was also used as a surrogate marker of treatment burden in stroke and cancer patients [41, 42] where a higher number of medications indicated increased burden of treatment. Open ended questions or linear analog indicators of burden were also used by some groups [43–45] with questions such as “Overall, how much are you bothered by any treatment related difficulties” or “the treatment so far was…” with responses ranging from not burdensome [1] to extremely burdensome [10].

Twelve studies described the assessment of treatment burden in patients with multiple chronic conditions. A variety of assessment tools were used with each reflecting the complexity of managing multiple chronic conditions by asking about many aspects of burden. Despite this shared purpose, these assessment instruments varied in content and length. Five studies used the Treatment Burden Questionnaire (TBQ) which rates fifteen items which may be associated with treatment burden on a 0–10 scale [11, 46–49]. The Multimorbidity Treatment Burden Questionnaire (MTBQ) is a concise (10 item) measure of treatment burden designed specifically for patients with multimorbidity [50]. The authors recommend its use in clinical practice to highlight specific problem areas for patients with multimorbidity such as problems with medications or lifestyle changes. The Willingness to Accept Life-Sustaining Treatment (WALT) instrument was used to examine, among other things, how treatment burden and treatment outcomes influence patient preferences across a wide range of chronic conditions [34]. Another group used a 78-item measure, the Patient Experience with Treatment and Self-management (PETS) measure. This explores many aspects of treatment burden including learning about health conditions, medications, appointments, monitoring health, exercise, diet, equipment, interpersonal challenges, expenses, healthcare providers, difficulty with services, social limitations, and exhaustion [51]. The Living with Medicines Questionnaire uses a Likert scale to rate 41 questions covering eight domains of medication burden [52]. Exercise is recommended as part of the management of many chronic conditions and its burden can be measured using the 10-item Exercise Therapy Burden Questionnaire [53]. The Health Care Task Difficulty (HCTD) scale assesses 8 different health care tasks [54]. The National Health and Aging Trends Study (NHATS) brief measure of treatment burden fielded in 2012 asked participants to reflect on the things they are asked to do to stay healthy or treat health problems (e.g. managing medicines, getting tests done, watching weight and blood pressure) [55, 56]. Some measures initially developed for chronic conditions were later adapted for specific conditions e.g. PETS for the diabetic population [57]. Additional detail on the instruments used to assess treatment burden are in Table 2 and Additional file 1.

#### Assessment of treatment burden experienced by caregivers

Six studies described assessing treatment burden experienced by caregivers through qualitative interview [19, 22, 25] and assessment tools (HCTD, TBQ) [7, 56] and survey [56]. Giovannetti used the HCTD scale to describe caregiver’s difficulty in assisting multimorbid older adults with eight health care tasks and found that difficulty increased with both advancing age of the caregiver and the number of healthcare tasks for which they were providing assistance [7]. Higher HCTD scores were associated with increased caregiver strain and depressive symptoms. Representatives from consumer health organizations representing individuals with chronic conditions highlighted the frustration and distress experienced by caregivers from treatment burden, citing examples of social isolation, deteriorating health, self-neglect, lack of support and marginalization resulting from the burden of their family member’s treatment [22]. In a study of individuals with chronic conditions and their caregivers [25], financial burden was the most widely discussed burden arising from treatment of chronic illness even in the Australian setting with federally provided universal health care. Caregivers described feelings of guilt caused by prioritizing the cost of medication over the broader needs of their family. Travelling with the person they cared for to access health services was also reported to be particularly burdensome for caregivers, mainly due to the logistics of transportation and parking falling to them. The importance of financial burden was echoed in recent studies on patient reported treatment burden in both Australia [27] and also in fee for service systems such as the United States where the burden is likely to be even higher [20].

In another study of the treatment burden experienced by people with chronic conditions [56] a quarter of participants with chronic conditions were also unpaid caregivers to another individual with a chronic condition. In this NHATS population having an unpaid caregiver predicted a substantial increase in patient reported treatment burden, however, being both a patient and carer did not have an effect on treatment burden. The authors hypothesized that this seemingly paradoxical effect could potentially be due to the differing psychological effects with care recipients experiencing feelings of guilt for burdening a family member while caregivers may feel a sense of self-worth and satisfaction while caring for a loved one. Other studies [19] also referred to the positive effects of caregiving describing caregiver participation in the treatment workload as practically onerous but an affirmation of the strength of the caregiver’s relationship with the patient. Despite this many caregivers reported feeling compelled to take on a caregiving role and described balancing treatment workload with their everyday life as being extremely demanding and limiting.

## Discussion

We performed a systematic literature review to identify articles assessing the treatment burden experienced by patients and caregivers to inform the feasibility of developing a quality indicator in this area for home-based medical care. We found no guidelines for healthcare professionals on when to assess for treatment burden and identified no one standardized assessment method. Qualitative data outlined the complexity of treatment burden as not just the burden of using a specific treatment, but all the adaptations and changes that need to be made to an individual’s daily life in order to successfully use a particular treatment. Very little data about caregiver treatment burden were identified.

Many instruments are available to assess treatment burden. Little or no work has been done to assess or compare the performance properties of the different instruments, so deciding which one to use is largely dependent on the number of comorbid conditions, the nature of the conditions and the time available for administration. Most currently available tools are disease-specific but have the potential to be adapted to other disease states, such as the Treatment Well-Being component of the Cystic Fibrosis Questionnaire-Revised (CFQ-R). As the majority of older adults now live with more than one condition [60] finding a useful tool to assess the burden of treatment in those living with multiple chronic conditions such as the Multimorbidity Treatment Burden Questionnaire [50] becomes increasingly important.

Surrogate measures of treatment burden, such as polypharmacy or medication regimen complexity are easier to extract from medical records but only capture one aspect of medication burden and therefore may be less useful to assess overall treatment burden. For example, a person with newly diagnosed hypertension has to manage not only possible polypharmacy, but what medication to take and when, side effects of the medication, follow-up monitoring and healthcare visits, insurance paperwork, lifestyle and dietary changes as well as the challenge of finding time to incorporate these changes into their already busy life. The burden only increases as the number and complexity of conditions increases and can make adherence challenging. For people with multiple chronic conditions, this is particularly important, and assessments of the multiple domains of treatment burden are vital. Although instruments such as PETS provide a very detailed assessment, they are time consuming to complete. A simple tool that is quick to administer such as the NHATS measure or the MTBQ may serve as an ideal screen to identify treatment burden. A screening question such as those used in the qualitative interviews or the ICAN Discussion Aid which is designed to take more than three minutes to administer, if positive could be followed by a more detailed assessment using the TBQ, MTBQ or the PETS.

Although most patients and healthcare providers are aware of treatment burden, the lack of a simple method to measure it in the clinical setting may lead to a lack of focus on this important aspect of patient centered care. Healthcare providers need to identify patients burdened by their treatment and engage in approaches to ameliorate it. One approach described in the literature is to engage in minimally disruptive medicine by tailoring treatment regimens to the realities of the daily lives of patients and relevant patient goals [10]. Patients also need education to encourage them to acknowledge their burden and talk to their provider about what can be done to reduce burden and help foster adherence.

Despite the extensive literature on caregiving burden relatively little information was available about treatment burden experienced by caregivers [7, 22, 25, 56]. Consumer health organization representatives pointed to the frustration and distress treatment burden causes for caregivers. This is especially true of caregivers of older adults with multiple chronic conditions, dementia or serious illness where social isolation, self-neglect, lack of support and marginalization are frequently experienced. Approximately 43 million Americans provide some type of ongoing, unpaid assistance to a family member or friend with a chronic illness or disability [61] with an estimated economic value of $450 billion [62]. Clearly, caregivers carry a significant burden related to the treatment of their family members which healthcare providers and society need to urgently acknowledge and address.

Strengths of this work include the systematic and comprehensive nature of the literature review that included both the peer-reviewed and grey literature and review of both quantitative and qualitative methods for assessing treatment burden. The limitations of our review largely reflect the shortcomings of the field and the lack of standardized tools and measures of treatment burden. The possibility that our search terms and multiple overlapping searches of both the peer-reviewed and grey literature failed to identify a relevant paper or source of information is low but remains a possibility especially as many authors refer to disease burden and treatment burden interchangeably. We chose to include both qualitative and quantitative studies in our review. We acknowledge the potential for qualitative studies to introduce bias by reporting on specific populations that may not be generalizable, however, we feel that they are under-utilized in systematic reviews and in this review are essential to illustrate the breadth of experiences and complexity of treatment burden [63].

## Conclusions

The burden of treatment for chronic conditions is a complex and significant issue for both patients and their caregivers. The predicted growth of the older population over the next few decades will increase the number of people living with multiple chronic conditions. Assessment tools for treatment burden need to be standardized and utilized and appropriate measures developed for different care settings including home care. Patients and caregivers need education in this critical area to help them advocate for care aligned with their individual goals and preferences. Physicians and the healthcare system need to work together to identify patients burdened by their treatment and develop individualized plans in partnership with patients and their families to address the burden.

## Additional file

Additional file 1: Details of population studied and instruments used to assess treatment burden. (DOCX 19 kb)

## Data Availability

The datasets used and/or analysed during the current study are available from the corresponding author on reasonable request.

## Abbreviations

CFQ-R: Cystic Fibrosis Questionnaire-Revised
CINAHL: The Cumulative Index to Nursing & Allied Health
COPD: Chronic Obstructive Pulmonary Disease
HCTD: Health Care Task Difficulty
ICAN: Instrument for Patient Capacity Assessment
MESH: Medical Subject Headlines used for indexing articles in PubMed
MTBQ: Multimorbidity Treatment Burden Questionnaire
NHATS: National Health and Aging Trends Study
NLM: National Library of Medicine
PETS: Patient Experience with Treatment and Self-management
PhD: Doctor of Philosophy
PRISMA: Preferred Reporting Items for Systematic Reviews and Meta-Analyses
QOL-B: Quality of Life-Bronchiectasis
TBQ: Treatment Burden Questionnaire
WALT: Willingness to Accept Life-Sustaining Treatment
