# Supplementary material for: A systematic literature review of the assessment of treatment burden experienced by patients and their caregivers
Source: BMC Geriatr. 2019 Oct 11;19:262. doi: 10.1186/s12877-019-1222-z (PMC6788093; doi:10.1186/s12877-019-1222-z)
Supplement: Supplementary file 1 — Details of population studied and instruments used to assess treatment burden. (DOCX 19 kb) [file 12877_2019_1222_MOESM1_ESM.docx]

Additional file 1: Table S1 Details of population studied and instruments used to assess treatment burden.

| **Study** | **Disease** | **Patient, caregiver or both** | **Question to assess treatment burden** |
| --- | --- | --- | --- |
| 65 | Asthma | Patient | Assessed burden of asthma medication using following questions as part of the Satisfaction with Inhaled Asthma Treatment Questionnaire:   - My asthma inhaler(s) fits easily in my purse or pocket - Carrying my asthma inhaler(s) can be cumbersome (e.g. size/weight) - I wish my asthma medication was easier to take - I sometimes leave home with the wrong inhaler - Using more than one inhaler can be a hassle or a problem - Using my asthma inhaler(s) in public is embarrassing |
| 35 | Bronchiectasis | Patient | QOL-B 3.0 questionnaire (1 of 8 scales)   - To what extent do your treatments for bronchiectasis make your daily life more difficult? - How much time do you currently spend each day on your treatments for bronchiectasis? - How difficult is it for you to fit in your treatments for bronchiectasis each day? |
| 58 | Cancer | Patient | How much are you bothered by any treatment related difficulties? 0 (not at all) to 100 (severely) |
| 45 | Cancer (breast) | Patient | As part of a quality of life questionnaire patients were asked:   - Overall how much are you bothered by any treatment related difficulties |
| 41 | Cancer (Lung) | Patient | Calculated number of encounter days (inpatient or outpatient) and defined high treatment burden as having total encounter days in the top quartile |
| 40 | Cancer (seminoma) | Patient | Treatment burden measured according to the number of treatment episodes with more episodes equating to higher burden. |
| 44 | Celiac disease | Patient | Participants asked to rate 4 domains on a scale of 0-100: difficulty in following treatment, perceived importance of following treatment, disease-specific health and overall health. |
| 34 | Cancer, congestive heart failure or chronic obstructive pulmonary disease | Patient | Willingness to Access life-Sustaining Treatment (WALT) instrument |
| 38 | Cystic Fibrosis | Patients | As part of the CFQ-R, treatment burden was assessed with 3 questions:  To what extent do your treatments make your daily life more difficult?  How much time do you currently spend each day on your treatments?  How difficult is it for you to do your treatments each day |
| 52 | Chronic conditions | Patient | Living with Medicines Questionnaire V3 (LMQ-3): 41-Likert-type statements based on a 5-point scale within 8 domains (Practical difficulties, perceived effectiveness, communication/relationships with HCPs, cost-related burden, concerns about medicine use, side effect burden, interference to day-to-day life, autonomy/control). Accompanied by a visual analogue scale that allows self-reporting of overall perceived burden and a free-text question allowing respondents to add further details of their medicine use experiences. |
| 53 | Chronic conditions | Patient | Exercise Therapy Burden Questionnaire: 10 item questionnaire measuring the burden of exercise therapy to treat chronic conditions:   - The exercises cause me pain - The exercises cause me fatigue - I get bored when I exercise (too much repetition, not enough fun). - The exercises in my program are too difficult. - I waste too much time exercising. - Exercising reminds me of my condition. - I lack support to exercise. - I lack motivation to exercise. - My exercises are not adapted to my physical activity objectives. - I feel that exercising is not efficient in my case. |
| 11, 46-49 | Multiple Chronic Conditions | Patient | Treatment Burden Questionnaire. Rate the following items from 0 (Not a problem) to 10 (Big problem)   - Problems related to taste, shape or size of tablets and/or the annoyances caused by injections - Number of times medication is taken daily - Efforts made not to forget to take medications - Necessary precautions when taking medication - Lab tests and other exams - Self-monitoring - Doctor visits and other appointments - Relationships with healthcare providers - Arranging medical appointments and reorganizing schedule around these appointments - Administrative burden related to healthcare - Financial burden associated with their healthcare - Burden related to dietary changes - Burden related to doctor’s recommendations to practice physical activity - Impact of healthcare on their relationship with others - Need for medical healthcare on a regular basis reminds them of their health problems |
| 7, 54 | Multimorbidity | Caregiver | Caregiver’s Health Care Task Difficulty (HCTD).  Caregivers were asked to rate the following tasks according to whether they did them and if they did them with no, some or a lot of difficulty:  Obtaining medications, planning medication schedule, administering medications, deciding to change medications, managing medical bills, scheduling patient appointments, arranging transportation, getting information, following recommended diet, monitoring patient’s health, obtaining medical equipment or obtaining community services. |
| 51  57 | Multimorbidity  Diabetes | Patient  Patient | Patient Experience with Treatment and Self-management (PETS). Multi-item scales assessing burden of chronic illness treatment and self-care as it relates to nine domains: medical information, medications, medical appointments, monitoring health, interpersonal challenges, medical/healthcare expenses, difficulty with healthcare services, role/social activity limitations, and physical/mental exhaustion. |
| 59 | Multimorbidity | Patient | Adapted version of TBQ to include financial burden question and side effects of medication question |
| 50 | Multimorbidity | Patient | A 10-item measure of treatment burden for patients with multimorbidity. Participants were asked to rate how much difficulty they have with the following:   1. Taking lots of medications 2. Remembering how and when to take medication 3. Collecting prescription medication 4. Monitoring your medical conditions (eg, checking your blood pressure or blood sugar, monitoring your symptoms, etc) 5. Arranging appointments with health professionals 6. Seeing lots of different health professionals 7. Attending appointments with health professionals (eg, getting time off work, arranging transport, etc) 8. Obtaining clear and up-to-date information about your condition 9. Making recommended lifestyle changes (eg, diet and exercise) 10. Having to rely on help from family and friends |
| 37 | Diabetes | Patient | Diabetes Treatment Burden Questionnaire (DTBQ), patient-administered questionnaire using 18 questions to measure treatment burden caused by pharmacotherapy for Type 2 diabetes. |
| 43 | Lupus nephritis | Patient | 2 questions on treatment burden:   1. “The treatment so far was….” On a 5 point Likert scale ranging from (1) not burdensome to (5) extremely burdensome. 2. What aspect of the treatment did you experience as burdensome? (Open-ended) |
| 56 | Medicare beneficiaries | Patient | Participants were asked to reflect on the things they are asked to do to stay healthy or treat health problems (managing medicines, getting tests and lab work done, watching weight and blood pressure, or having yearly exams) using 4 questions:  • “How often are these things difficult for you to do?”  • “How often are these things difficult for your family or close friends to handle?”  • “How often do these things that you do to stay healthy or treat health problems get delayed or not get done?”  • “How often do you feel that doctors or other providers ask you to do too much?” |
| 38 | Psoriasis | Patient | Dermatology Life Quality Index (10 item questionnaire, 4 possible responses to each question, score range 0-30, higher scores = higher impact on quality of life). One question on treatment burden   - Over the last week, how much of a problem has the treatment for your skin been, for example by making your home messy, or by taking up time? |
| 42 | Stroke | Patient | Polypharmacy used as a measure of treatment burden |
| 39 | Urinary Incontinence | Patient | Patient Satisfaction Questionnaire: 51 questions rated on a 5 point Likert scale – a subset of questions used to identify treatment burden. |

QOL: Quality Of Life; CHQ: Child health questionnaire; CFQ-R: Cystic Fibrosis Questionnaire – Revised; TBQ: Treatment Burden Questionnaire; Caregiver’s Health Care Task Difficulty: HCTD; Patient Experience with Treatment and Self-management: PETS; Living with Medicines Questionnaire V3: LMQ-3
